# Hepatitis C virus infection of cholangiocarcinoma cell lines

**DOI:** 10.1099/vir.0.000090

**Published:** 2015-06

**Authors:** Nicola F. Fletcher, Elizabeth Humphreys, Elliott Jennings, William Osburn, Samantha Lissauer, Garrick K. Wilson, Sven C. D. van IJzendoorn, Thomas F. Baumert, Peter Balfe, Simon Afford, Jane A. McKeating

**Affiliations:** ^1^​ Centre for Human Virology, Viral Hepatitis Laboratory, University of Birmingham, Birmingham B15 2TT, UK; ^2^​ Centre for Liver Research, University of Birmingham, Birmingham B15 2TT, UK; ^3^​ Department of Medicine, Johns Hopkins Medical Institutions, Baltimore, MD, USA; ^4^​ Department of Cell Biology, University Medical Center Groningen, University of Groningen, Groningen 9713AV, The Netherlands; ^5^​ Inserm U1110, University of Strasbourg 3 Rue Koeberlé, F-67000 Strasbourg, France

## Abstract

Hepatitis C virus (HCV) infects the liver and hepatocytes are the major cell type supporting viral replication. Hepatocytes and cholangiocytes derive from a common hepatic progenitor cell that proliferates during inflammatory conditions, raising the possibility that cholangiocytes may support HCV replication and contribute to the hepatic reservoir. We screened cholangiocytes along with a panel of cholangiocarcinoma-derived cell lines for their ability to support HCV entry and replication. While primary cholangiocytes were refractory to infection and lacked expression of several entry factors, two cholangiocarcinoma lines, CC-LP-1 and Sk-ChA-1, supported efficient HCV entry; furthermore, Sk-ChA-1 cells supported full virus replication. *In vivo* cholangiocarcinomas expressed all of the essential HCV entry factors; however, cholangiocytes adjacent to the tumour and in normal tissue showed a similar pattern of receptor expression to *ex vivo* isolated cholangiocytes, lacking SR-BI expression, explaining their inability to support infection. This study provides the first report that HCV can infect cholangiocarcinoma cells and suggests that these heterogeneous tumours may provide a reservoir for HCV replication *in vivo*.

## Introduction

Hepatitis C virus (HCV) is an enveloped positive strand RNA virus classified in the genus *Hepacivirus* of the family *Flaviviridae*. Worldwide, approximately 170 million individuals are persistently infected with HCV that leads to a progressive liver disease, including cirrhosis and hepatocellular carcinoma (reviewed by Scheel & Rice, 2013). The major cell type in the liver supporting HCV replication is hepatocytes (Kandathil *et al.*, 2013; Wieland *et al.*, 2014). Hepatocytes and cholangiocytes derive from a common epithelial progenitor cell that proliferates during liver inflammation (Roskams, 2006); however, to date there are no published studies investigating the permissivity of cholangiocytes to support HCV infection.

Cholangiocarcinomas account for approximately 10 % of all primary hepatic cancers and can be classified as intrahepatic or extrahepatic in location (Patel, 2006; Roskams, 2006). Cholangiocarcinomas are heterogeneous and can arise from cholangiocytes, liver progenitor cells (Komuta *et al.*, 2012) or hepatocytes (Fan *et al.*, 2012; Sekiya & Suzuki, 2012). HCV is a known risk factor for cholangiocarcinoma, together with hepatitis B virus and other chronic inflammatory conditions (Patel, 2006; Ralphs & Khan, 2013).

Primary cholangiocytes isolated from donor liver tissue, along with a panel of cholangiocarcinoma derived cell lines, were screened for their ability to support HCV entry and replication. Primary cholangiocytes were refractory to HCV entry or replication, however, two cholangiocarcinoma cell lines supported efficient HCV entry. Furthermore one of the tumour lines, Sk-ChA-1, supported HCV entry and replication at comparable levels to primary human hepatocytes. Cholangiocarcinoma tumours expressed all of the essential HCV entry factors, whereas biliary epithelia lacked expression of one essential entry receptor, scavenger receptor BI (SR-BI). In summary, we demonstrate that a subset of cholangiocarcinomas support HCV replication, consistent with reports showing HCV RNA in intrahepatic cholangiocarcinomas (Lu *et al.*, 2000; Perumal *et al.*, 2006; Yin & Chen, 1998), highlighting a potential new reservoir that merits further investigation.

## Results

### Cholangiocarcinomas support HCV pseudotype particle (HCVpp) entry

**Fig. 1.**
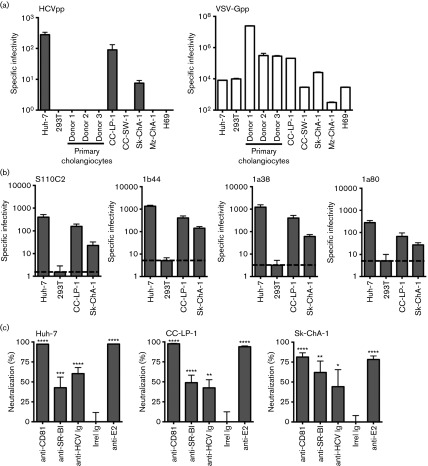
HCVpp infects cholangiocarcinoma cell lines. (a) Primary cholangiocytes or cholangiocarcinoma cell lines, along with control permissive (Huh-7 hepatoma) and non-permissive (293T) cells, were infected with HCVpp (strain H77) or VSV-Gpp, and infection levels expressed relative to the no-envelope control. (b) Infectivity of HCVpp expressing primary envelope glycoproteins (Osburn *et al.*, 2014) for CC-LP-1 and Sk-ChA-1 along with Huh-7 and 293T control cells. (c) Anti-receptor antibodies (anti-CD81 or SR-BI), anti-HCV Ig or anti-E2 (3/11) inhibition of HCVpp infection of CC-LP-1, Sk-ChA-1 and control Huh-7 cells. Data are presented relative to control antibody. *N* = 3 independent experiments. ****P<0.0001, ***P<0.001, **P<0.01, *P<0.05.

To investigate the ability of cholangiocytes to support HCV entry we screened primary cells isolated from 10 donor liver explants with various disease aetiologies, together with cell lines derived from normal tissue (H69), intra- (CC-LP-1 and CC-SW-1) and extra-hepatic cholangiocarcinomas (Sk-ChA-1 and Mz-ChA-1). We confirmed that all of the cholangiocarcinoma derived lines expressed the epithelial markers EpCAM, CK19 and epithelial membrane antigen NCAM and GCTM-5. HCVpp failed to infect any of the primary cholangiocytes, however, we observed efficient entry into two cell lines, CC-LP-1 and Sk-ChA-1 (Fig. 1a). All cell lines tested supported vesicular stomatitis virus G pseudotype particle (VSV-Gpp) entry, demonstrating functional lentiviral promoter activity in these cells (Fig. 1a). To investigate whether cholangiocarcinoma cells supported the entry of HCVpp expressing diverse envelope glycoproteins, we generated pseudoparticles expressing E1E2 glycoproteins cloned from HCV genotype 1a/b acutely infected subjects (Osburn *et al.*, 2014). As controls we included the well-characterized Huh-7 hepatoma line and the non-permissive claudin-1 null human embryonic kidney 293T cell line. All HCVpp strains infected CC-LP-1 and Sk-ChA-1 cholangiocarcinoma lines with comparable efficiency to Huh-7 but failed to infect 293T (Fig. 1b). We confirmed that CC-SW-1 and Mz-ChA-1 cells were refractory to all patient-derived HCVpp (data not shown). To investigate the receptor dependency of HCVpp infection of the permissive cholangiocarcinoma lines, we assessed the ability of anti-CD81 and anti-SR-BI antibodies to inhibit HCVpp (strain H77) infection. Both antibodies inhibited HCVpp entry, demonstrating receptor-dependent entry (Fig. 1c). In addition, anti-E2 (3/11) and polyclonal IgG purified from chronic-HCV infected subjects inhibited HCVpp infectivity (Fig. 1c) but had no effect on VSV-Gpp infection (data not shown). These studies show that some tumour-derived cholangiocytes but not those isolated from non-tumour liver tissue support HCVpp entry.

### Cholangiocarcinoma express HCV entry factors

**Fig. 2.**
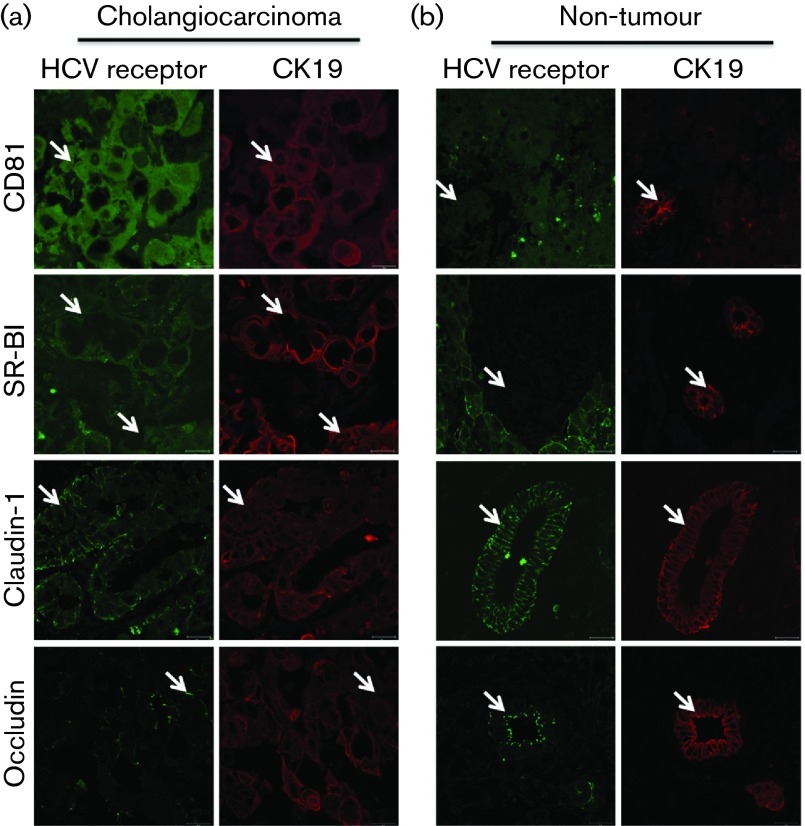
Cholangiocarcinoma expresses HCV entry factors. (a) Cholangiocarcinoma and (b) normal non-tumour margin tissue was stained (arrows) with antibodies specific for HCV receptors (CD81, SR-BI, claudin-1 and occludin) (green) and epithelial marker CK19 (red). A representative donor tissue is shown, where arrows denote dual CK19/receptor expressing cells. Scale bars represent 20 µm.

**Fig. 3.**
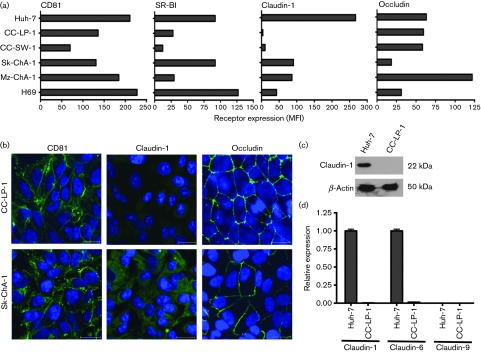
Cholangiocarcinoma *in vivo* expresses HCV entry factors (a) Flow cytometry data of HCV receptor expression in cholangiocarcinoma cells and control Huh-7 hepatoma cells. Expression levels are expressed as Mean Fluorescent Intensity (MFI) relative to species-specific control antibodies. (b) Confocal microscopic images of HCV receptors in permissive CC-LP-1 and Sk-ChA-1 cells. Scale bars represent 20 µm. (c) Claudin-1 expression in Huh-7 and CC-LP-1 cells analysed by Western blotting. (d) Real-time quantitative reverse-transcription PCR (qRT-PCR) analysis of claudin-1, -6 and -9 mRNA expression in Huh-7 and CC-LP-1 cells.

To investigate HCV entry factor expression *in vivo* we stained cholangiocarcinoma liver tissue from two donors with antibodies specific for CD81, SR-BI, claudin-1, occludin and epithelial marker CK19. Cholangiocarcinoma from both donors expressed all four HCV entry factors, albeit with low CD81 expression (Fig. 2a), whereas biliary epithelia from the normal non-tumour margin lacked SR-BI expression (Fig. 2b). To assess whether the cholangiocarcinoma cell lines show a similar profile of receptor expression to the tumour tissue, the cells were stained for receptor expression along with Huh-7 hepatoma cells as a positive control. The permissive cell line Sk-ChA-1 expressed all four entry factors at comparable levels to Huh-7 hepatoma cells (Fig. 3a). Of note, CC-LP-1 cells expressed CD81, SR-BI and occludin; however, we failed to detect any claudin-1 expression (Fig. 3a). Both permissive cell lines expressed CD81 and occludin at the plasma membrane; however, claudin-1 was predominantly intracellular in Sk-ChA-1 cells and not observed in CC-LP-1 cells (Fig. 3b). The two non-permissive cholangiocarcinoma lines, CC-SW-1 and Mz-ChA-1, expressed low levels of SR-BI, similar to that observed for biliary epithelia in non-tumour liver tissue, suggesting that this may be the limiting factor for HCV entry. These data show that cholangiocarcinoma and epithelial cells isolated from the tumour express all four HCV entry receptors, consistent with their permissivity to support HCV entry.

### Cholangiocarcinoma CC-LP-1 express negligible claudin-1, -6 and -9 and yet support HCV entry

Several studies have reported that HCV can use several members of the claudin family to infect cells, including claudin-1, -6 and -9 (Meertens *et al.*, 2008; Zheng *et al.*, 2007). Western blot analysis for claudin-1 expression confirmed our earlier confocal images and flow cytometry data that claudin-1 was undetectable in CC-LP-1 cells (Fig. 3c). Furthermore, we failed to detect claudin-1, -6 and -9 mRNA in CC-LP-1 cells (Fig. 3d). As expected, Huh-7 expressed high levels of claudin-1 and -6 mRNA but minimal claudin-9, consistent with previous reports (Zheng *et al.*, 2007).

**Fig. 4.**
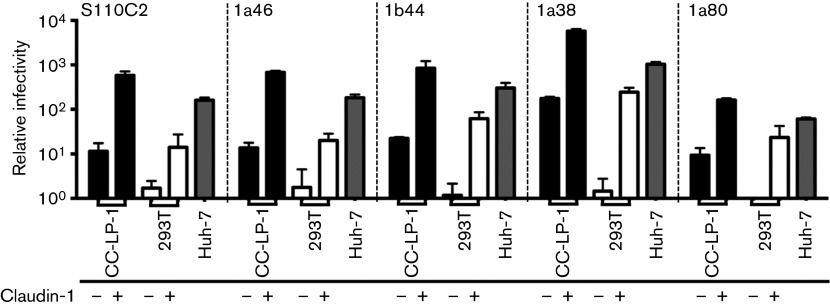
Claudin-1 expression in CC-LP-1 cells promotes HCVpp infection. CC-LP-1 (black) and claudin-null 293T (white) cells were transduced to express claudin-1 and inoculated with HCVpp expressing a range of envelope glycoproteins. Huh-7 hepatoma cells (grey) were included as a control. Claudin-1 expression promoted HCVpp infection of 293T and CC-LP-1 cells. Only CC-LP-1 supported HCVpp infection in the absence of claudin-1 overexpression. *N* = 3 independent experiments.

To determine whether HCVpp expressing patient-derived envelope glycoproteins required claudin-1 to initiate infection we assessed their ability to infect the claudin-null cell line, 293T, and CC-LP-1 cells following claudin-1 overexpression. Claudin-1 expression was confirmed by flow cytometry (data not shown). HCVpp strains only infected 293T cells expressing claudin-1, demonstrating claudin-1 dependent entry. However, the same viruses infected parental CC-LP-1 cells, however, their infection levels were increased following claudin-1 expression in this cellular background (Fig. 4). In summary, diverse HCVpp strains infect CC-LP-1 cholangiocarcinoma cells independent of claudin-1, -6 or -9 expression.

### Cholangiocarcinoma Sk-ChA-1 support HCV entry and genome replication

**Fig. 5.**
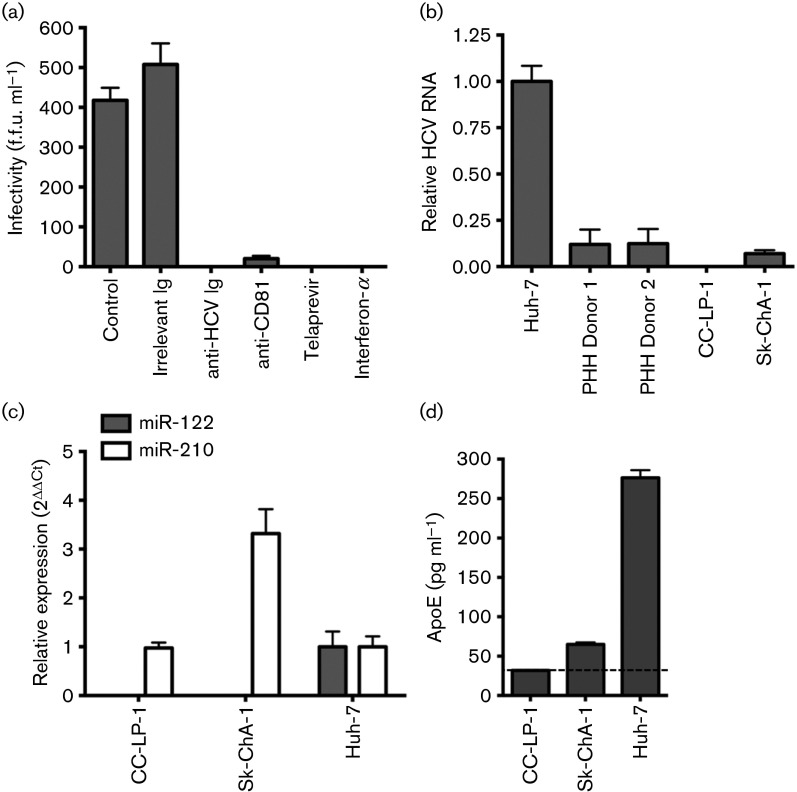
HCVcc infection of cholangiocarcinoma cells. (a) Sk-ChA-1 cells were inoculated with HCVcc strain SA13/JFH (titre of 10^6^ IU ml^−1^ based on Huh-7 cells) in the presence or absence of anti-HCV Ig (100 µg ml^−1^), anti-CD81 antibody (clone 2s131, 10 µg ml^−1^), protease inhibitor Telaprevir (1 µg ml^−1^) and interferon-α (10 IU ml^−1^). Data are presented as f.f.u. ml^−1^ calculated as NS5A antigen-expressing cells. (b) Sk-ChA-1, but not CC-LP-1, cells support HCV SA13/JFH RNA replication at comparable levels to PHH. HCV RNA levels were normalized to Huh-7 cells. (c) Cholangiocarcinoma cells do not express miR-122 but control miR-210 was detected. (d) Sk-ChA-1 cells secrete low levels of ApoE, whereas we failed to detect ApoE from CC-LP-1 cells, where the dotted line indicates the ELISA cut-off point. *N* = 3 independent experiments.

Sk-ChA-1 cells support cell-culture-derived HCV (HCVcc) (strains SA13/JFH-1 and JFH-1) replication as assessed by enumerating NS5A expressing cells, whereas CC-LP-1 failed to express detectable levels of viral antigen or RNA. Similar results were obtained with CC-LP-1 cells transduced to express claudin-1, suggesting that their non-permissivity to support HCVcc replication was not due to a claudin-1 independent viral uptake pathway. Foci of NS5A expressing Sk-ChA-1 only comprised on average 2–4 cells, suggesting minimal viral spread. Infection was inhibited by polyclonal patient IgG from pooled HCV-infected donors, neutralizing anti-CD81, Telaprevir (VX-950) and interferon-α (Fig. 5a). To compare the permissivity of Sk-ChA-1 cells to support HCV replication to primary human hepatocytes (PHHs) we measured HCV RNA levels 72 h post-infection and included permissive Huh-7 cells as a positive control. We noted comparable levels of HCV RNA in Sk-ChA-1 and PHHs from two donors (Fig. 5b). Huh-7 cells supported significantly higher levels of HCV replication, most likely explained by their negligible Toll-like receptor 3 (TLR3) expression and limited ability to sense replicating RNA (Wang *et al.*, 2009). Attempts to infect the highly permissive Huh-7.5 cell line with extracellular virus secreted from Sk-ChA-1 or PHHs cells failed to establish infection, most likely due to the low frequency of infected cells and comparable to the life cycle observed in infected neuroepithelioma cells (Fletcher *et al.*, 2010).

Several cellular factors have been demonstrated to facilitate HCV infection and replication, including miR-122 (Jopling *et al.*, 2005), Niemann-Pick C1-like 1 (NPC1L1) cholesterol absorption receptor (Sainz *et al.*, 2012) and Apolipoprotein E (ApoE) (Jiang & Luo, 2009). We therefore investigated the expression of these factors in the two cholangiocarcinoma cell lines. Neither CC-LP-1 nor Sk-ChA-1 cells expressed detectable levels of miR-122 (Fig. 5c). Both cell lines expressed NPC1L1 at similar levels to Huh-7 cells, as measured by Western blotting (data not shown), suggesting that neither of these reported host-cell factors explained the differential permissivity of the cholangiocarcinoma cells to support HCVcc infection. In contrast, we noted a significant difference in ApoE expression between the two lines, with the permissive Sk-ChA-1 secreting detectable levels of ApoE (Fig. 5d). These results demonstrate that Sk-ChA-1, but not CC-LP-1, cholangiocarcinoma cells support HCV replication.

## Discussion

CC-LP-1 and Sk-ChA-1 cells derived from intra- and extra-hepatic cholangiocarcinoma tissue, respectively, support HCV entry or replication. In contrast, HCV did not infect primary cholangiocytes. Infection of cholangiocarcoma cells was inhibited by antibodies specific for CD81, SR-BI, E2 glycoprotein and by pooled immunoglobulin from chronic HCV infected patients. Of note, CC-LP-1 expressed negligible levels of tight junction protein, claudin-1 mRNA or protein and yet supported the entry of HCVpp expressing a range of patient-derived envelope glycoproteins. CCl-LP-1 cells do not express detectable levels of claudin-6 or -9 mRNA suggesting that infection is claudin-independent. We confirmed that the infectivity of all HCVpp strains tested were claudin-1 dependent in 293T cells and expression of claudin-1 in CC-LP-1 cells significantly enhanced HCVpp entry. To the best of our knowledge this is the first report of claudin-1 independent HCV entry into hepatic derived epithelial cells and supports a model where CD81 and SR-BI mediate viral attachment and internalization in the absence of claudin-1, -6 or -9 co-expression.

Cholangiocytes and hepatocytes arise from a common progenitor cell type, termed oval cells in rodents and reactive ductular cells in humans (Roskams, 2006). During chronic liver diseases, reactive ductular cells become activated and differentiate into cholangiocytes and hepatocytes, depending on the nature of the liver injury. Cholangiocarcinomas develop from cholangiocytes and reactive ductular cells, however, recent studies provide evidence that intrahepatic cholangiocarcinoma can also originate from hepatocytes (Fan *et al.*, 2012; Sekiya & Suzuki, 2012). This occurs through activation of NOTCH and AKT signalling in hepatocytes, leading to the conversion of hepatocytes into cholangiocytes with concurrent malignant transformation (Fan *et al.*, 2012). These data may explain why viral hepatitis is a risk factor for cholangiocarcinoma, since infected hepatocytes can undergo NOTCH activation and lineage conversion (Iwai *et al.*, 2011; Sekiya & Suzuki, 2012).

We did not assess the ability of purified hepatic progenitor cells to support HCV infection, however, NCAM and EpCAM-positive cholangiocytes isolated from donor livers with primary biliary cirrhosis are known to contain an enriched population of reactive ductular cells, and were refractory to infection. In addition, primary cholangiocytes isolated from subjects with primary sclerosing cholangitis, a condition that predisposes to cholangiocarcinoma, were refractory to infection (Roskams *et al.*, 1990) (E. Gershwin, personal communication). CC-LP-1 and CC-SW-1 cells are derived from intrahepatic cholangiocarcinomas (Shimizu *et al.*, 2006) whereas Sk-ChA-1 and Mz-ChA-1 are derived from extrahepatic tumours of the biliary tree and gall bladder, respectively (Knuth *et al.*, 1985), indicating that the ability to support HCV replication is not restricted to intrahepatic cholangiocarcinomas.

Viral tropism is defined at multiple levels of the virus life cycle, including entry, RNA replication and assembly (reviewed by Scheel & Rice, 2013). In addition to the four essential factors for HCV entry, CD81, SR-BI, claudin-1 and occludin, several additional factors facilitate infection. These include epidermal growth factor receptor (EGFR) (Lupberger *et al.*, 2011), Niemann-Pick C1-like 1 cholesterol absorption receptor (Sainz *et al.*, 2012) and the liver-specific micro-RNA, miR-122 (Jopling *et al.*, 2005). Virus particles are secreted in association with apolipoproteins and ApoE expression enhances viral infectivity and virus particle production (Da Costa *et al.*, 2012; Hueging *et al.*, 2013; Jiang & Luo, 2009). Sk-ChA-1, but not CC-LP-1, cells supported HCV replication that was inhibited by interferon-α, anti-CD81, HCV-positive pooled patient IgG and Telaprevir. While the levels of infection observed in Sk-ChA-1 cells was significantly lower than Huh-7 cells, Sk-ChA-1 cells supported similar levels of infection to PHHs. Huh-7 cells have previously been shown to support significantly higher HCV replication than PHH, likely due to reduced sensing of HCV RNA and a lack of TLR3 expression in these cells (Farquhar & McKeating, 2008; Wang *et al.*, 2009). Since Sk-ChA-1 but not CC-LP-1 cells support HCV replication, we quantified the expression of additional host factors reported to facilitate infection to explore the differences in these cell lines to replicate HCVcc. None of the cell lines expressed miR-122, similar to our previous observations with blood–brain barrier endothelial cells and HepG2-CD81 hepatoma cells that support low level HCVcc replication (Fletcher *et al.*, 2012; Israelow *et al.*, 2014). Of note, only Sk-ChA-1 cells secrete detectable levels of ApoE, which, together with their expression of the essential HCV entry factors, may explain their permissivity to support HCV replication.

In summary, we have identified two cell lines derived from cholangiocarcinoma tissue that support efficient HCV entry and low-level HCV replication. Our data raises the possibility that cholangiocarcinomas may represent a reservoir for HCV infection *in vivo* and warrant further studies to establish the role of HCV in cholangiocarcinoma pathogenesis.

## Methods

### 

#### Cells and reagents.

Huh-7 and 293T HEK cells were provided by C. Rice (Rockefeller University) and cholangiocarcinomas (CC-LP-1, CC-SW-1, Mz-ChA-1 and Sk-ChA-1) by P. Bosma (University of Amsterdam). Cells were maintained in Dulbecco's modified Eagle's medium (DMEM) supplemented with 10 % FBS, 1 % non-essential amino acids and 1 % penicillin/streptomycin. H69 cells derived from normal intrahepatic biliary epithelia were cultured as previously reported (Grubman *et al.*, 1994). Human hepatocytes were isolated according to previously published protocols (Mitry, 2009) and maintained in Williams E medium with 10 % FBS/5 mM HEPES/insulin/dexamethasone. Primary cholangiocytes were isolated from end stage liver disease tissue and ethical permission was granted by the local research ethics committee (CA/5192; Research Ref. 06/Q702/61). Briefly, liver (~30 g) was diced and incubated with collagenase type 1A (Sigma). The digest was layered onto a 33 % and 77 % Percoll gradient and centrifuged at 500 ***g*** for 30 min. The interface layer was collected, washed three times in PBS, and incubated with a cholangiocyte-specific mAb specific for HEA 125 (Progen). Cholangiocytes were positively selected by incubating with anti-mouse IgG1-coated Dynabeads (Invitrogen) and by magnetic separation. The cells were cultured in DMEM, Hams F12, 10 % heat-inactivated human serum, 1 % penicillin/streptomycin and glutamine, HGF (10 ng ml^−1^, Peprotech), EGF (10 ng ml^−1^, Peprotech), cholera toxin (10 ng ml^−1^, Sigma), tri-iodo-thyronine (2 nM, Sigma), hydrocortisone (2 µg ml^−1^) and insulin (0.124 IU ml^−1^). In all experiments, cells were used between passage two and five to ensure phenotypic stability.

The following primary antibodies were used: anti-CD81 (clone 2s131); (in house); anti-SRBI (gift from Pfizer); anti-claudin-1 (R&D Technologies); anti-occludin (Invitrogen); anti-NS5A-9E10 (C. Rice, Rockefeller University, NY); and anti-CK19 (Vector Laboratories). Secondary antibodies used were: Alexa 488 goat anti-rabbit immunoglobulin IgG; Alexa 488 goat anti-mouse IgG; and Alexa 594 goat anti-mouse IgG (Invitrogen).

#### Liver tissue and confocal imaging.

Formalin fixed and paraffin embedded biopsies were obtained from patients with cholangiocarcinoma that was diagnosed according to standard biochemical and histological criteria: all tissues studied were selected by an experienced histopathologist. Liver sections (10 µm) were deparaffinized and rehydrated in water followed by low temperature antigen retrieval. Sections were blocked with 2 % Caesin (Vector Laboratories) and incubated with anti-CD81, anti-SR-BI, anti-claudin-1 and anti-occludin along with anti-CK19. Bound antibodies were detected with Alexa-conjugated secondary anti-species antibodies and labelled sections mounted using Fluorescent Imaging Media (Dako). Images were acquired using an upright Zeiss 780 laser scanning confocal microscope (100×1.4NA objective), where microscope settings were optimized for each fluorescent protein to obtain the highest signal to noise ratio whilst controlling for cross talk. Background fluorescence intensities were determined from the fluorescent signal of an Ig isotype control.

#### Flow cytometry.

Cell surface receptor expression was monitored by live-cell staining and flow cytometry as previously reported (Fletcher *et al.*, 2012). Briefly, cells were incubated with anti-SR-BI, anti-CD81 or anti-claudin-1 for 1 h. To detect occludin cells were fixed and permeabilized followed by incubation with a primary antibody. After a brief wash the cells were labelled with a fluorescent conjugated secondary antibody for 1 h. Thereafter, cells were fixed with 1 % paraformaldehye (Sigma) and data collected using a FACS calibur flow cytometer (BD Biosciences) and analysed with FlowJo software (Tree Star).

#### HCVpp and HCVcc genesis and infection.

Pseudoparticles were generated by transfecting 293T cells with plasmids encoding a human immunodeficiency virus (HIV) provirus expressing luciferase and vesicular stomatitis virus G (VSV-G), a panel of HCV envelope glycoproteins (Dowd *et al.*, 2009) or a no-envelope control, as previously reported (Hsu *et al.*, 2003). Supernatants were harvested at 48 h post-transfection, clarified and filtered through a 0.45 µm membrane. Virus-containing medium was added to target cells plated in 96-well plates seeded at 5×10^5^ cells cm^−2^. At 72 h post-infection, cells were lysed and luciferase activity measured in a luminometer (Lumat LB 9507). HCVpp infectivity was calculated by expressing the HCV or VSV-G luciferase signal (relative light units, RLU) relative to the No env RLU value. HCVcc NS5A-positive foci were enumerated and infectivity expressed as f.f.u. ml^−1^.

To generate HCVcc, plasmids encoding chimeric SA13/JFH (Jensen *et al.*, 2008) or J6/JFH-1 (Lindenbach *et al.*, 2005) were used to generate HCV RNA as previously described (Lindenbach *et al.*, 2005). Briefly, RNA was electroporated into Huh-7.5 cells, supernatants collected at 72 and 96 h and stored at −80 °C. Various cell lines and PHHs were inoculated with HCVcc for 6 h in the presence or absence of anti-HCV Ig (100 µg ml^−1^), anti-CD81 (2s131 at 10 µg ml^−1^), Telapravir (1 µg ml^−1^) or interferon-α (10 IU ml^−1^). Unbound virus was removed by washing and the cells re-fed fresh media plus or minus antiviral agents and propagated for 72 h before fixing for NS5A detection or extraction of total cellular RNA for HCV RNA quantification. Cells were fixed with ice-cold methanol and stained for NS5A with mAb 9E10 and an isotype-matched Alexa 488-conjugated anti-mouse IgG2a.

#### Neutralization of HCV infection.

Huh-7, CC-LP-1, Sk-ChA-1 or 293T cells were seeded in 96-well plates at 5×10^5^ cells cm^−2^. The cells were incubated 24 h post-seeding with 10 µg ml^−1^ anti-receptor or irrelevant IgG control mAb. After 1 h, HCV-H77pp, VSV-Gpp or No-envpp, or HCVcc, was added and incubated for 72 h at 37 °C. In addition, anti-E2 mAbs or HCV^+^ IgG was incubated with virus for 1 h prior to infecting the appropriate target cells. At 72 h post-infection, luciferase activity was measured for HCVpp infections, or cells stained for NS5A. The percentage neutralization was calculated relative to the irrelevant IgG control.

#### Transduction of cells to express claudin-1.

CC-LP-1 or 293T cells were transduced to express claudin-1 as previously described (Flint *et al.*, 2006; Harris *et al.*, 2010). Briefly, packaged lentiviruses to express claudin-1 were generated by cotransfection of 293T cells with plasmids encoding VSV-G protein, HIV Gag-Pol, and pTRIP-claudin-1 (1 : 3:3 ratio). Cells were seeded at 4×10^5^ cells cm^−2^ and infected 24 h later with the packaged lentivirus. After 12 h, cells were seeded into appropriate plates either for HCVpp or HCVcc infection as described.

#### Real-time reverse transcriptase PCR.

RNA was prepared using the Qiagen RNeasy or MiRNeasy kit for microRNA analysis. Purified cellular RNA samples were amplified for HCV RNA (Primer Design Ltd), claudin-1, -6 or -9, or miR-122 in a quantitative reverse-transcription PCR (qRT-PCR) in accordance with the manufacturer’s guidelines (CellsDirect kit; Invitrogen) using an ABI7500 PCR machine (Applied Biosystems). Glyceraldehyde 3-phosphate dehydrogenase (GAPDH) or miR-210 were included as endogenous controls for amplification efficiency, and HCV amplification normalized to GAPDH using the ΔΔCt method.

#### ApoE ELISA.

Cells were cultured for 24 h and supernatant harvested. Secreted ApoE levels were measured using a commercial ApoE ELISA (Abcam) according to the manufacturer’s instructions.

#### Statistical analysis.

Results are expressed as the mean±1 standard deviation of the mean. Statistical analyses were performed using Student's *t*-test in Prism 6.0 (GraphPad) with a *P*<0.05 being considered statistically significant.
